# UPLC-Orbitrap-MS/MS Combined With Biochemical Analysis to Determine the Growth and Development of Mothers and Fetuses in Different Gestation Periods on Tibetan Sow Model

**DOI:** 10.3389/fnut.2022.836938

**Published:** 2022-03-29

**Authors:** Longmiao Zhang, Chengquan Tan, Zhongquan Xin, Shuangbo Huang, Junwu Ma, Meiyu Zhang, Gang Shu, Hefeng Luo, Baichuan Deng, Qingyan Jiang, Jinping Deng

**Affiliations:** ^1^Guangdong Provincial Key Laboratory of Animal Nutrition Control, National Engineering Research Center for Breeding Swine Industry, Institute of Subtropical Animal Nutrition and Feed, College of Animal Science, South China Agricultural University, Guangzhou, China; ^2^State Key Laboratory of Pig Genetic Improvement and Production Technology, College of Animal Science and Technology, Jiangxi Agricultural University, Nanchang, China; ^3^College of Animal Science and Technology, Guangdong Polytechnic of Science and Trade, Guangzhou, China; ^4^Dekon Food and Agriculture Group, Chengdu, China

**Keywords:** gestation periods, mothers, fetuses, serum, amniotic fluid

## Abstract

Pregnancy is a complex and dynamic process, the physiological and metabolite changes of the mother are affected by different pregnancy stages, but little information is available about their changes and potential mechanisms during pregnancy, especially in blood and amniotic fluid. Here, the maternal metabolism rules at different pregnancy stages were investigated by using a Tibetan sow model to analyze the physiological hormones and nutrient metabolism characteristics of maternal serum and amniotic fluid as well as their correlations with each other. Our results showed that amniotic fluid had a decrease (*P* < 0.05) in the concentrations of glucose, insulin and hepatocyte growth factor as pregnancy progressed, while maternal serum exhibited the highest concentrations of glucose and insulin at 75 days of gestation (*P* < 0.05), and a significant positive correlation (*P* < 0.05) between insulin and citric acid. Additionally, T4 and cortisol had the highest levels during late gestation (*P* < 0.05). Furthermore, metabolomics analysis revealed significant enrichment in the citrate cycle pathway and the phenylalanine/tyrosine/tryptophan biosynthesis pathway (*P* < 0.05) with the progress of gestation. This study clarified the adaptive changes of glucose, insulin and citric acid in Tibetan sows during pregnancy as well as the influence of aromatic amino acids, hepatocyte growth factor, cortisol and other physiological indicators on fetal growth and development, providing new clues for the normal development of the mother and the fetus, which may become a promising target for improving the well-being of pregnancy.

## Introduction

Pregnancy is one of the most critical periods for mothers and fetuses, the huge flow of physiological changes and metabolic adaptations it involves may have adverse consequences in different stages of pregnancy (1). Specifically, in early pregnancy, undernutrition retards embryonic growth and results in symmetrically small and low-birth-weight fetuses (2, 3). In mid and late pregnancy periods, improper maternal physiological adaptation or abnormal endocrine function may cause pregnancy complications, such as gestational diabetes, fetal growth restriction, fetal overgrowth, abnormal amniotic fluid, etc. (4). In late pregnancy, placental dysplasia or dysfunction is generally considered to be the main cause of preeclampsia (5, 6), leading to premature birth and even death (7, 8). Additionally, ambient air pollution (9), age, parity (10), etc., will also affect maternal and fetal health. With better understanding the laws of maternal metabolism at different stages of pregnancy, even small improvements in obstetric healthcare can improve the well-being of many women and children.

The various biochemical indicators in maternal blood can not only reflect maternal health to a certain extent, but also show the growth and development of the fetus. During the entire gestation period, nutrients are transported to the endometrium through the mother’s blood, and then to the fetus *via* the placenta, with placenta as the main organ to transfer nutrients between mother and fetus (11). Amniotic fluid is the internal environment for fetal growth and development in the uterus. In early pregnancy, before skin keratinization occurs, the fetus mainly exchanges nutrients through skin and amniotic fluid, and after skin keratinization, the fetus can also swallow amniotic fluid to take in nutrients, bioactive factors, and the synthetic precursors of antibacterial substances to promote its growth and development (12). However, few studies have been performed on the changing laws of maternal blood and amniotic fluid and the related potential mechanisms at different gestation periods.

Pigs are increasingly used as human metabolic disease models due to their similarity in metabolic features, cardiovascular system, and proportional organ sizes (13). The purpose of the present study was to reveal the nutritional needs and metabolic changes in the growth and development of mothers and fetuses by biochemical analysis, metabolome analysis and correlation analysis of the serum and amniotic fluid samples collected from Tibetan sows at different gestation periods. This work not only provides a reference for regulating the pregnancy process, but also guides the direction for normal fetal growth and development.

## Materials and Methods

The study was approved by the Animal Ethics Committee of South China Agricultural University, and all the experiments followed the guidelines of Guangdong Province on the Review of Welfare and Ethics of Laboratory Animals.

### Sample Source and Collection

In this study, 12 Tibetan healthy primiparous sows were selected for experiment, and they were allowed to have the same diet twice a day and free access to drinking water during gestation. The 12 sows were divided into four groups according to their gestation time: 33, 50, 75, and 90 days of gestation (coded as G33, G50, G75, and G90, respectively). All samples were collected from each group as follows: maternal blood samples were collected from the ear veins of sows with anticoagulant blood collection tubes. After standing for 0.5 h, the blood was centrifuged at 5,000 × *g* at 4°C for 10 min to separate the serum, followed by collecting the serum aliquots and storage at −80°C for further analysis.

After blood collection, the sows were anesthetized by an intravenous (i.v.) injection with ketamine/xylazin (15 mg/kg Ursotamin and 1.5 mg/kg Xylazin, Serumwerk Bernburg, Bernburg, Germany), followed by slaughter through heart bloodletting, collecting the entire uterus by hysterotomy, and extracting amniotic fluid from the intact amniotic sac using a syringe (14). Finally, the amniotic fluid samples were centrifuged at 5,000 × *g* at 4°C for 10 min to collect the supernatant and stored at −80°C for further analysis.

Collectively, blood samples were collected separately on 33, 50, 75, and 90 days of gestation from G33, G50, G75, and G90 groups (*n* = 3), and amniotic fluid was sampled separately on day 33, 50, and 75 from G33, G50, and G75 groups (*n* = 3). Each sample was analyzed in duplicate with three biological replicates.

### Determination of Biochemical Parameters and Related Physiological Hormones

After thawing the test samples at 4°C, the concentrations of glucose, total protein (TP) and non-esterified fatty acids (NEFA) in the serum and amniotic fluid were measured by the microplate method using the commercial kits (Nanjing Jiancheng Bioengineering Institute, Nanjing, China). Insulin, insulin-like growth factor 1 (IGF-1), hepatocyte growth factor (HGF), transforming growth factor β (TGF-β), basic fibroblast growth factor (bFGF), cortisol, T3 and T4 in serum and amniotic fluid were analyzed using commercial ELISA kits, with T3 ELISA kits purchased from Wuhan Huamei Biological Engineering (Wuhan, China), and all the other assay kits from Jiangsu Meimian Industrial Co., Ltd., (Zhangjiagang, China). All kits were used as instructed by the manufacturers.

### Analysis of Serum and Amniotic Fluid Metabolome

#### Sample Preparation

After thawing, the serum and amniotic fluid samples were vortexed for 2 min to full homogenization, followed by mixing 200 μL of each sample in a 1.5 mL centrifuge tube with 800 μL methanol (Merck, Darmstadt, Germany) to remove proteins. After mixing for 2 min under vortexing, the mixture was centrifuged at 4°C and 14,500 rpm for 15 min to collect the supernatant. Next, 1,000 μL of the supernatant was placed in a 1.5 mL centrifuge tube, followed by centrifugation in vacuum for 2 h, and blowing dry with nitrogen gas at room temperature. After adding 400 μL of methanol-water (1:1, v/v), the mixture was extracted in an ultrasonic bath for 15 min at room temperature, followed by centrifugation at 4°C and 14,500 rpm for another 15 min, and transferring 200 μL of the supernatant to sample vials for UPLC–Orbitrap–MS/MS analysis (15, 16).

### UPLC–Orbitrap–MS/MS Analysis

The experiments were performed on a Thermo Fisher Scientific UPLC system (Dionex UltiMate 3000), and Xcalibur software (version 3.0) was used for instrument control, data acquisition and data analysis. Chromatographic separation of all samples was performed on a C_18_ Hypersil Gold (100 mm × 2.1 mm, 1.9 μm, Thermo Scientific, Waltham, MA, United States) column under the following conditions: column temperature, 35°C; flow rate, 0.2 mL/min; mobile phase, ultrapure water-0.1% formic acid solution (Sigma, St. Louis, MO, United States) (eluent A) and acetonitrile (Merck, Darmstadt, Germany) (eluent B); gradient program settings, 0–3 min, 5–7% B; 3–5 min, 7–13% B; 5–15 min, 13–50% B; 15–18 min, 50–5% B, finally, equilibrated with 5% B for 2 min; total running time, 20 min; injection volume, 2 μL. The mass spectrometry was performed in the positive and negative ionization modes, and data were obtained from electrospray ionization (ESI) under following conditions: ion source, ESI source; spray voltage, 4 kV (−4 kV in ESI-), 3.5 kV (+3.5 kV in ESI+); heater temperature, 300°C; atomizing gas (sheath gas) pressure, 40 bar; auxiliary gas pressure, 10 bar; transfer capillary temperature, 320°C; MS scanning mode, full scan; resolution and in-source collision induced dissociation (in-source CID), 35,000 and 0 V, respectively. MS/MS scanning mode was data dependent ms2scan (dd-ms2), with a resolution of 17,000 and high collision induced dissociation (HCD) energy of 10, 30, and 50 eV (15, 16).

### Statistical Analyses

The data of biochemical parameters/physiological hormones in the serum and amniotic fluid of Tibetan sows at different gestation periods were analyzed using the general linear model procedure (SPSS, 20.0). The test results were subjected to a one-way analysis of variance (ANOVA), and the significant difference was determined by the Duncan’s multiple-range test at *P* < 0.05. Tamhane’s T2 test was used to assess variance heterogeneity. All results are expressed as mean values ± standard error of mean (SEM) (*n* = 3). Additionally, Xcalibur (Thermo Fisher, Waltham, MA, United States) was used to acquire the raw metabolome data, MZmine 2.2.1 was allowed to extract accurate masses and peak areas, then the exact mass combined with MS/MS spectrum was matched with online mass databases. Normalized the obtained data for more intuitive and reliable results, followed by clustering analysis of the obtained differential metabolites, using the Kyoto Encyclopedia of Genes and Genomes (KEGG) database^1^ to determine the most important biochemical metabolic pathways and signal transduction pathways involved in the metabolites. Finally, Spearman’s correlation analysis was performed to test the relationship between biochemical parameters, related physiological hormones and differential metabolites using the OmicStudio tools,^2^ with significant differences considered at *P* < 0.05.

## Results

### Biochemical Parameters in the Serum and Amniotic Fluid of Tibetan Sows at Different Gestation Periods

**FIGURE 1:**
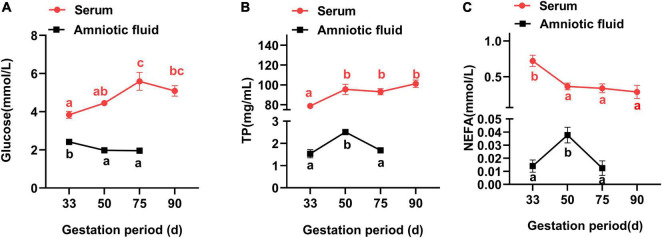
Changes of biochemical parameters in the serum and amniotic fluid of Tibetan sows at different gestation periods. **(A)** Glucose; **(B)** TP; and **(C)** NEFA. Data are expressed as mean ± SEM (*N* = 3). TP, total protein; NEFA, non-esterified fatty acids. Different lowercase letters represent significant difference at *P* < 0.05.

In Figure 1, the maternal serum was seen to increase (*P* < 0.05) in the glucose concentration from G33 to G75, but with no obvious difference between G75 and G90 (*P* > 0.05). Additionally, the concentration of TP was lower (*P* < 0.05) at G33 than at G50, G75 or G90, in contrast to an opposite trend for the NEFA concentration (*P* < 0.05). Meanwhile, the amniotic fluid showed a higher concentration of glucose at G33 than at G50 or G75 (*P* < 0.05), with higher TP and NEFA at G50 than at G33 or G75 (*P* < 0.05).

### Physiological Hormones in the Serum and Amniotic Fluid of Tibetan Sows at Different Gestation Periods

**FIGURE 2:**
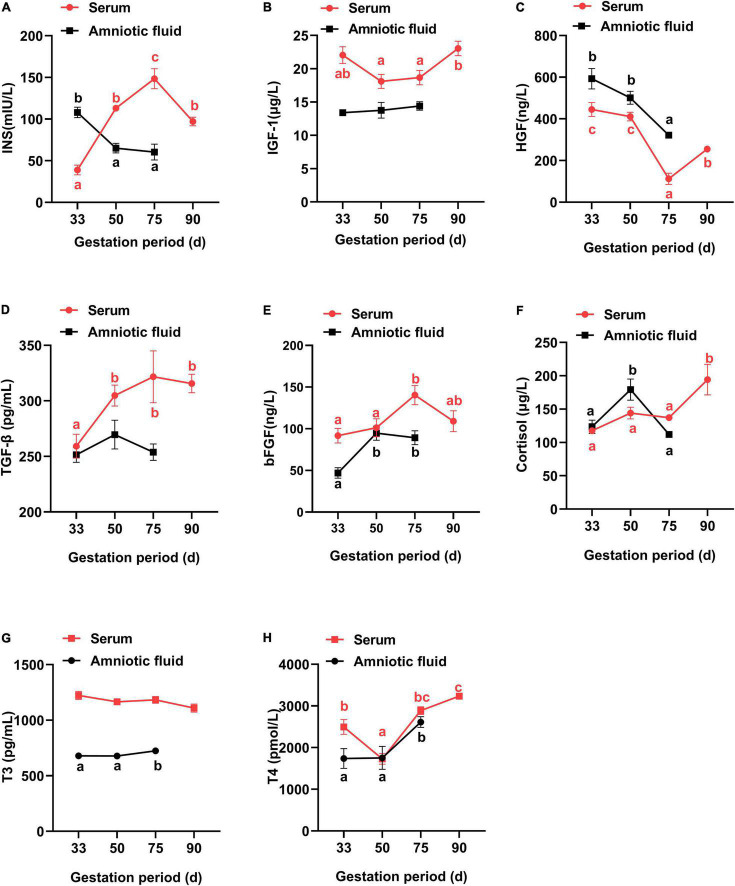
Changes of physiological hormones in the serum and amniotic fluid of Tibetan sows at different gestation periods. **(A)** INS; **(B)** IGF-1; **(C)** HGF; **(D)** TGF-β; **(E)** bFGF; **(F)** Cortisol; **(G)** T3; **(H)** T4. Data are expressed as mean ± SEM (*N* = 3); INS, insulin; IGF-1, insulin-like growth factor 1; HGF, hepatocyte growth factor; TGF-β, transforming growth factor β; bFGF, basic fibroblast growth factor; T3, triiodothyronine; T4, thyroxine. Different lowercase letters represent significant difference at *P* < 0.05.

In Figure 2, the maternal serum showed an increase in the insulin concentration from G33 to G75, but failed to further increase at G90, with a lower insulin concentration at G90 than at G75 (*P* < 0.05). Additionally, both IGF-1 and T4 had the lowest concentration at G50 (HGF at G75), while bFGF showed the highest concentration at G75 (*P* < 0.05). Meanwhile, both TGF-β and cortisol had higher concentrations in late gestation (G90) than at early gestation (G33) (*P* < 0.05), and the concentration of T3 showed no obvious change at different gestation periods (*P* > 0.05).

For amniotic fluid, IGF-1 and TGF-β showed no obvious changes at different gestation periods (*P* > 0.05). Additionally, the concentrations of T3 and T4 were increased from G33 to G75, in contrast to a decrease for insulin and HGF (*P* < 0.05). Furthermore, bFGF and cortisol had higher concentrations at G50 than at G33, respectively (*P* < 0.05).

### Serum Metabolome Analysis of Tibetan Sows at Different Gestation Periods

**FIGURE 3:**
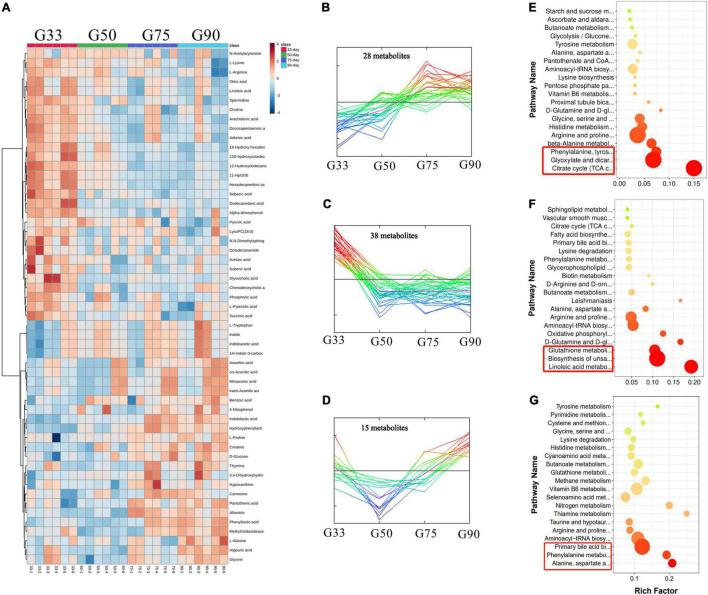
Analysis of serum metabolome in Tibetan sows at different gestation periods. **(A)** Heatmap of the major differential metabolites in the serum of Tibetan sows at different gestation periods; **(B–D)** Cluster analysis of differential metabolites; **(E–G)** The enriched KEGG pathways for each cluster.

A total of 107 differential metabolites were identified in the serum of Tibetan sows at different gestation periods (Figure 3), and cluster analysis showed 28 of them increased with the progress of gestation, with the first three enriched pathways including citrate cycle (TCA cycle), glyoxylate dicarboxylate metabolism, and phenylalanine/tyrosine/tryptophan biosynthesis (Figures 3B,E). Additionally, 38 differential metabolites had the highest concentrations at G33, with the enriched pathways including linoleic acid metabolism and the biosynthesis of unsaturated fatty acids and glutathione metabolism (Figures 3C,F). Furthermore, 15 differential metabolites were found to have the lowest levels at G50, with the enriched pathways in the decreasing order of alanine/aspartate/glutamate metabolism, phenylalanine metabolism, and primary bile acid biosynthesis (Figures 3D,G).

### Correlation Between Biochemical Parameters/Physiological Hormones and Differential Metabolites in Serum at Different Gestation Periods

**FIGURE 4:**
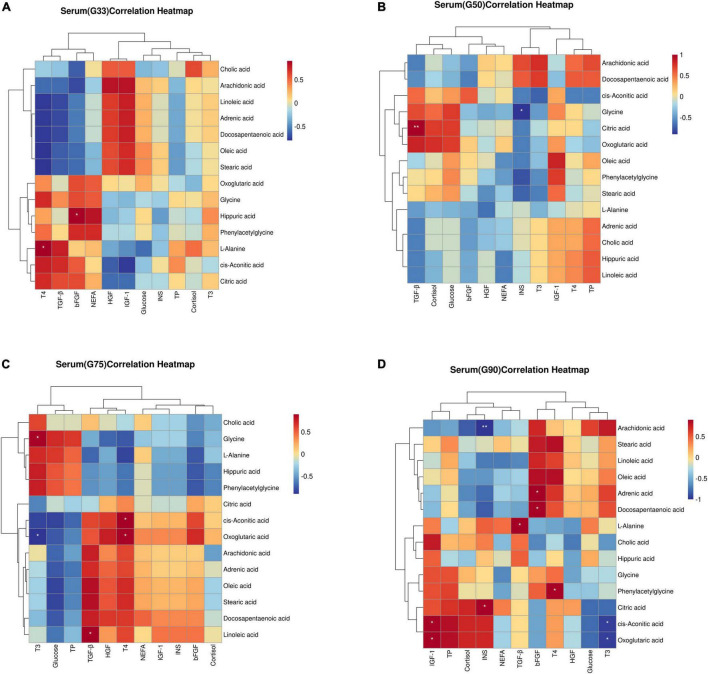
Correlation between biochemical parameters/physiological hormones and differential metabolites in serum at different gestation stages: **(A)** G33; **(B)**G50; **(C)**G75; **(D)** G90. The correlation coefficient is represented by different colors (red, positive correlation; blue, negative correlation). *represents significantly negative or positive correlations (*P < 0.05 and **P < 0.01).

Spearman correlation analysis was performed to evaluate the potential relationship between biochemical parameters/physiological hormones and differential metabolites at different gestation periods (Figure 4). At G33, T4 was significantly positively correlated with L-Alanine (*P* < 0.05) (Figure 4A). At G50, insulin was significantly negatively correlated with glycine (*P* < 0.05), while TGF-β was significantly positively correlated with citric acid (*P* < 0.01) (Figure 4B). At G75, T3 had a significant positive correlation with glycine, but a significant negative correlation with oxoglutaric acid (*P* < 0.05) (Figure 4C). At G90, insulin was significantly positively correlated with citric acid (*P* < 0.05), but significantly negatively correlated with arachidonic acid (*P* < 0.01). Additionally, T3 had a significant negative correlation with oxoglutaric acid and *cis-*aconitic (*P* < 0.05), while T4 showed a significant positive correlation with phenylacetylglycine (*P* < 0.05) (Figure 4D).

### Amniotic Fluid Metabolome Analysis of Tibetan Sows at Different Gestation Periods

**FIGURE 5:**
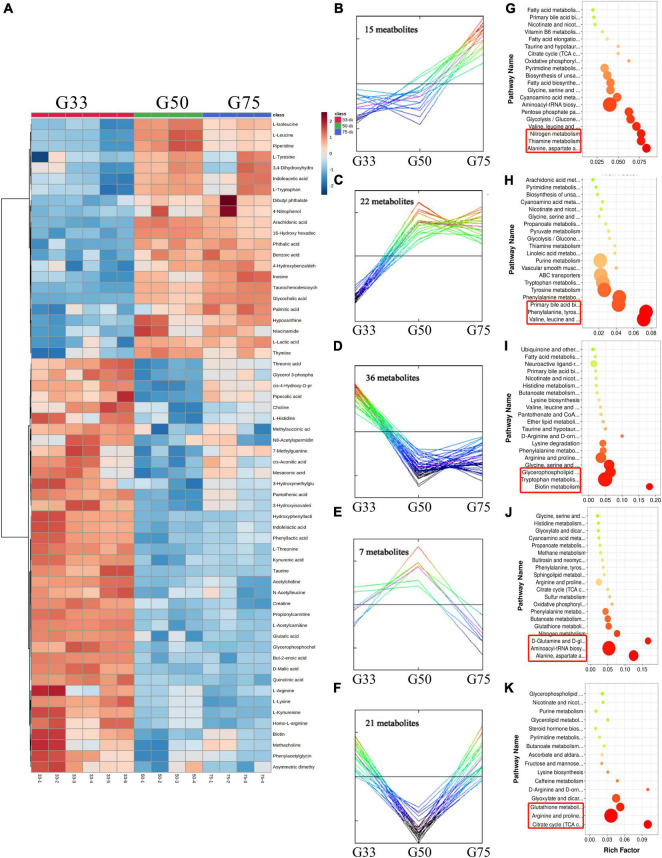
Amniotic fluid metabolome analysis of Tibetan sows at different gestation periods. **(A)** Heatmap of the major differential metabolites in the amniotic fluid of Tibetan sows at different gestation periods; **(B–F)** Cluster analysis of differential metabolites; **(G–K)** The enriched KEGG pathways for each cluster.

A total of 101 differential metabolites were found in the amniotic fluid of Tibetan sows at different gestation periods (Figure 5). Cluster analysis revealed 15 of the differential metabolites increased with the progress of gestation, with enriched pathways including alanine/aspartate/glutamate metabolism, thiamine metabolism and nitrogen metabolism (Figures 5B,G). Additionally, 22 differential metabolites showed continuous upregulation from G33 to G50, with the first three enriched pathways including valine/leucine/isoleucine biosynthesis, phenylalanine/tyrosine/tryptophan biosynthesis, and primary bile acid biosynthesis (Figures 5C,H). Meanwhile, 36 differential metabolites continued to be downregulated from G33 to G50, with enriched pathways including biotin metabolism, tryptophan metabolism, glycerophospholipid metabolism (Figures 5D,I). Moreover, seven differential metabolites had the turning point at G50, being up-regulated from G33 to G50 while down-regulated from G50 to G75, with enriched pathways including alanine/aspartate/glutamate metabolism, aminoacyl-tRNA biosynthesis, D-Glutamine and D-glutamate metabolism (Figures 5E,J). Furthermore, 21 differential metabolites also had the turning point at G50, with downregulation from G33 to G50 while upregulation from G50 to G75, and the enriched pathways mainly included TCA cycle, arginine/proline metabolism, and glutathione metabolism (Figures 5F,K).

### Correlation Between Biochemical Parameters/Physiological Hormones and Differential Metabolites in Amniotic Fluid at Different Gestation Periods

**FIGURE 6:**
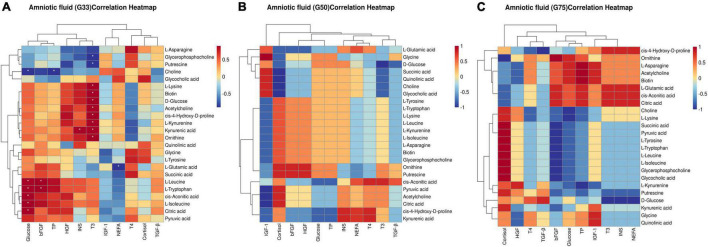
Correlation between biochemical parameters/physiological hormones and differential metabolites in amniotic fluid at different gestation stages: **(A)** G33; **(B)** G50; **(C)** G75. The correlation coefficient is represented by different colors (red, positive correlation; blue, negative correlation). *represents significant negative or positive correlations (*P < 0.05).

In the amniotic fluid collected at different gestation periods, correlation analysis showed that at G33, glucose had significant positive correlations with citric acid, L-leucine, L-isoleucine, L-tryptophan, and *cis-*aconitic acid (*P* < 0.05), and a significant negative correlation with choline (*P* < 0.05) (Figure 6A). Additionally, insulin was significantly positively correlated with kynurenic acid (*P* < 0.05), and bFGF was significantly positively correlated with L-leucine and L-tryptophan (*P* < 0.05) (Figure 6A). However, no significant correlation was detected between biochemical parameters/physiological hormones and differential metabolites in the amniotic fluid at G50 and G75 (Figures 6B,C).

## Discussion

In this study, we identified a variety of reproductive hormones and biochemical parameters in Tibetan sows’ serum and amniotic fluid, whose abundance altered during pregnancy progression. In addition, we performed untargeted metabolomics profiling and identified dynamic temporal regulation of metabolic changes during gestation of Tibetan sows: more than half of the measured metabolites and metabolic pathways changed during pregnancy. These parameter changes presumably reflect important maternal biological physiology during pregnancy and fetal growth, and may imply physiological advantages of Chinese *Tibetan pigs* over other pig breeds.

### Insulin Resistance Adaptive Response During Late Gestation

Glucose is an important nutrient in the control of fetal growth. Studies of diabetic women have shown that low blood glucose levels during pregnancy as a result of excessively tight glycemic control lead to a greater incidence of intrauterine growth retardation, whereas having high blood glucose levels contributes to a high incidence of macrosomia (17). In this study, the maternal glucose level began to decrease after the peak at G75, and the glucose level in amniotic fluid continued to decrease with the progress of gestation, which reflected the changes of maternal and fetal glucose. This is consistent with reports from other large livestock and humans that the mid and late stages of gestation are the window period for rapid fetal weight gain, when the fetus and placenta require large amounts of glucose uptake and utilization (18–20).

It is worth noting that the concentration of insulin also showed a decrease in late gestation in the current study. While in the late gestation period, in order to meet the nutritional demand for rapid fetal growth, the mother will adaptively produce insulin resistance (21–23). This can be attributed to the decreased sensitivity and responsiveness of peripheral insulin target organs to normal insulin levels, resulting in a decrease in the efficiency of glucose uptake and utilization and making the body compensatively secrete more insulin to maintain blood glucose stability. Previous studies have shown that persistent hyperglycemia (24) and concomitant hyperinsulinemia (25) during late gestation are typical signs of insulin resistance. Interestingly, our findings were in direct contrast with other studies about other high-yield sows (24–26), implying that the Tibetan sows might have no or limited insulin resistance in the late gestation period. We speculated that Tibetan pigs, as lean pigs, had higher insulin sensitivity or stronger glucose tolerance than other obese pig breeds, which can be supported by previous reports that lean pigs had higher insulin sensitivity and glucose tolerance than obese pigs (27, 28).

Meanwhile, citric acid or citrate was reported to promote liver mitochondrial biogenesis, lipid oxidation and energy consumption through the AMP-activated protein kinase (AMPK) signaling network to improve liver insulin sensitivity in mice, indicating a positive correlation between citric acid and insulin resistance (29). This was similar to our result that insulin was significantly positively correlated with citric acid at G90 and the citrate cycle was the first enriched pathway (the increase of differential metabolites in serum with advancing gestation) in late gestation (G90). Furthermore, the concentration of TCA cycle metabolites, especially citric acid, was reported to be higher in patients of non-alcoholic fatty liver disease with high insulin sensitivity than those with low insulin sensitivity (30). However, whether citric acid improves insulin sensitivity in late gestation still needs to be further verified.

### Placental Angiogenesis During Gestation

Placenta, a combination of the fetus and the mother, is an important guarantee for the fetus to obtain maternal nutrition (11). Placental angiogenesis is critical to the development and survival of fetal pigs (31). The density of blood vessels in the placenta of Tibetan sows was shown to increase with the progress of gestation in our recent study (unpublished data). In particular, the enriched pathways for differential metabolites in the serum, including biosynthesis of phenylalanine/tyrosine/tryptophan, also increased with advancing gestation, with the highest level in late gestation as revealed by metabolome analysis. This intriguing observation raised the question of whether phenylalanine, tyrosine and tryptophan are related to high blood vessel density in late gestation.

Most of phenylalanine in the body is converted to L-tyrosine by phenylalanine hydroxylase (PAH) (32) to synthesize important neurotransmitters and hormones together with tyrosine. Tyrosine is a precursor for the production of natural catecholamines, including dopamine, norepinephrine and epinephrine (33). Previous studies on tumor angiogenesis have shown that catecholamines, norepinephrine and epinephrine could enhance the expression of angiogenic cytokines, vascular endothelial growth factor (VEGF), matrix metalloproteases (MMPs) etc., to stimulate tumor angiogenesis (34–36). Furthermore, tumor cells and placental trophoblast cells were reported to possess very similar characteristics in biological behaviors, such as proliferation, invasion, and angiogenesis (37, 38). In a mouse knockout model study, the targeted deletion of α_2*B*_-adrenoceptor gene was shown to cause defective placental labyrinth angiogenesis, which may be related to the inhibition of α_2*B*_-adrenoceptor on the expression of antiangiogenic receptors in sponge trophoblast cells (39). Additionally, angiogenesis induced by the derivatives of tryptophan, such as serotonin (40), was mediated through the Gβγ-activated serotonin receptor-linked Src/PI3K/Akt/NOX pathway (41). These reports indicate that phenylalanine, tyrosine, tryptophan and their derivatives could significantly promote angiogenesis, which might be the strategy the Tibetan sows adopt in late gestation to regulate placental blood vessel density and meet the nutritional needs for rapid fetal growth.

### Fetal Muscle Development During Gestation

Pregnancy is a critical period for fetal muscle development. The number of muscle fibers in piglets is fixed at birth, and embryonic myoblasts were reported to fuse at G33 to form primary muscle fibers (42, 43). In early gestation, amniotic fluid was the main nutrition source for fetal growth and development. In the present study, the concentrations of amniotic fluid glucose, insulin and HGF were shown to be higher in early gestation than in the other gestation periods. HGF, also known as scatter factor (SF), could increase the number of myoblasts to the optimal myoblast density through mitogenic and chemotactic activity and then started to fuse (44). This process was mediated by the HGF/SF-met signaling pathway. HGF ligand was reported to bind to its receptor c-met tyrosine kinase and use the specific activities of receptor tyrosine residues to promote the expansion and morphological transformation of myoblasts (45, 46) through the corresponding signaling pathways, such as Ras-Ral GEF-Ral pathway (47), PI3K-Akt (48), mitogen-activated protein kinase (MAPK) (49), etc. Meanwhile, related studies have found that during mouse embryogenesis, met and HGF/SF transcripts were present at the site of muscle formation (50). Anastasi et al. further proved that the expressions of HGF/SF and met genes were up-regulated in the proliferation of myoblasts, followed by a gradual decrease after entering the differentiation phase (45). As a signal of nutrient availability, insulin could increase the body’s uptake of glucose (51) and also could regulate muscle protein synthesis, degradation and protein type conversion through the mammalian target of rapamycin (mTOR) signaling pathway (52). *In vitro* and *vivo* experiments unveiled that insulin perfusion could promote animal muscle protein synthesis and inhibit protein catabolism (53), thus contributing to muscle protein synthesis.

Additionally, serum cortisol concentration was shown to increase with the progress of gestation, probably because the cortisol concentration during gestation was affected by estrogen levels, leading to an increase in corticosteroid-binding globulin (CBG) levels, followed by a decrease in the cortisol catabolism in the liver and an increase in the half-life of cortisol (54).

The placenta is a barrier to prevent maternal cortisol from reaching the fetus. In mammals, only 10–20% of maternal cortisol could be passed to the fetus through the placenta, and 80–90% was transformed into an inert form during placental transmission (55). Meanwhile, amniotic fluid cortisol was mainly derived from the adrenal glands of the fetus (54), which represents the activity state of the fetal adrenal cortex function, and could be used as one of the prenatal diagnostic indicators for fetal growth and development. In the present study, we also found no significant difference in serum T3 concentration at different gestation periods, while a significant increase of T4 in late gestation, suggesting that fetal development may mainly depend on whether the mother has sufficient T4 content rather than T3 content.

## Conclusion

In summary, our results demonstrated the adaptive changes of Tibetan sows during pregnancy and their possible mechanisms, suggested the potential effects of aromatic amino acids such as phenylalanine, tyrosine and tryptophan in placental development, and explained the important roles of HGF, insulin, cortisol and T4 in the fetal growth process. This study provides useful information for promoting normal development of the mother and the fetus during pregnancy.

## Data Availability Statement

The original contributions presented in the study are included in the article/supplementary material, further inquiries can be directed to the corresponding authors.

## Ethics Statement

The animal study was reviewed and approved by the Animal Ethics Committee of South China Agricultural University.

## Author Contributions

LZ, CT, and ZX participated in the writing and editing of the article. SH, JM, MZ, and GS contributed to the study design. HL, BD, QJ, and JD revised the manuscript. All authors contributed to the article and approved the submitted version.

## Conflict of Interest

HL was employed by Dekon Food and Agriculture Group. The remaining authors declare that the research was conducted in the absence of any commercial or financial relationships that could be construed as a potential conflict of interest.

## Publisher’s Note

All claims expressed in this article are solely those of the authors and do not necessarily represent those of their affiliated organizations, or those of the publisher, the editors and the reviewers. Any product that may be evaluated in this article, or claim that may be made by its manufacturer, is not guaranteed or endorsed by the publisher.

## Footnotes

1
http://www.genome.jp/kegg/

2
http://www.omicstudio.cn/tool
